# Supplementary material for: Cytomegalovirus Immunoglobulin G Levels and Subclinical Arterial Disease among People Living with HIV in Botswana: A Cross-Sectional Study
Source: Biomedicines. 2024 Apr 23;12(5):935. doi: 10.3390/biomedicines12050935 (PMC11118051; doi:10.3390/biomedicines12050935)
Supplement: Supplementary file 1 [file biomedicines-12-00935-s001.zip › biomedicines-2883794-supplementary.pdf]

biomedicines

MDPI

Supplementary Materials

Table S1. Associations between CMV and baseline demographic factors by study group.

	**All Study participants (N=183)**		**HIV-negative controls (N=79)**		**HIV-infected Groups (N=104)**			
					**HIV-infected on ART (N=68)**		**HIV-infected ART naïve (N=36)**	
	**r* with CMV**	**p-value**	**r* with CMV**	**p-value**	**r* with CMV**	**p-value**	**r* with CMV**	**p-value**
**Age**	0.05	0.52	0.03	0.79	0.17	0.16	-0.02	0.93
**CD4 nadir**			N/A	N/A	-0.21	0.17	N/A	N/A
**CD4 current**			N/A	N/A	-0.06	0.62	-0.04	0.82
**ART duration**			N/A	N/A	0.16	0.19	N/A	N/A
			**Mean (SD) CMV**	**p-value****	**Mean (SD or 95% CI) CMV**	**p-value****	**Mean (SD) CMV**	**p-value****
**Gender**								
Male	64.1 (71.1)	0.81	54.7 (69.6)	0.56	53.2 (48.1)	0.34	115.2 (95.8)	0.22
Female	61.9 (58.1)		46.8 (44.4)		66.0 (60.1)		80.8 (70.6)	
**ART switch**								
0			N/A	N/A	55.1 (32.8,77.5)	0.37	N/A	N/A
1			N/A	N/A	55.6 (24.8,86.3)		N/A	N/A
> 2			N/A	N/A	79.1 (41.9,116.3)		N/A	N/A
**ART regimen**								
PI			N/A	N/A	78.9 (48.8,109.0)	0.11	N/A	N/A
NNRTI			N/A	N/A	54.2 (36.6,71.7)		N/A	N/A
**Waist-hip ratio**	0.10	0.20	0.09	0.45	0.11	0.39	0.003	0.98

**Cigarette smoking**								
No	61.9 (63.5)	0.56	49.4 (55.9)	0.61	59.2 (55.0)	0.78	91.6 (82.7)	0.48
Yes	69.8 (72.7)		58.0 (73.3)		65.9 (54.6)		123.1 (87.4)	
**Hypertension diagnosis**								
No	63.9 (66.0)	0.59	51.2 (59.3)	0.98	59.7 (56.3)	0.97	95.1 (82.6)	
Yes	55.6 (54.3)		50.7 (63.8)		60.4 (45.9)		--	
**HBA1C**	0.02	0.83	0.03	0.83	0.10	0.40	-0.23	0.22
**Non-HDL cholesterol**	0.03	0.71	0.21	0.06	0.05	0.66	-0.37	0.03
